# The effect of self-management education through weblogs on the quality of life of diabetic patients

**DOI:** 10.1186/s12911-019-0941-6

**Published:** 2019-10-29

**Authors:** Amal Mohammad Rasoul, Rostam Jalali, Alireza Abdi, Nader Salari, Mehrali Rahimi, Masoud Mohammadi

**Affiliations:** 1 Department of Nursing, School of Nursing and Midwifery, Kermanshah University of Medical Sciences, Kermanshah, Iran; 2 Diabetes Research Center, Kermanshah University of Medical Sciences, Kermanshah, Iran

**Keywords:** Training, Self-management, Weblog, Quality of life, Diabetes

## Abstract

**Background:**

Self-management education of diabetes which is one of the most important noncommunicable diseases worldwide involves facilitating knowledge, skills, and ability required for self-care in these patients. Concerning the progressive growth of use of Internet for educating patients and absence of different studies about education through use of weblogs in patients with diabetes in Iran, the present study was conducted with the aim of determining the effect of self-management education through weblogs on the quality of life of affect the patients.

**Methods:**

This study was performed as intervention on patients referring to diabetes clinic of Talghani hospital in Kermanshah in winter 2018 and spring 2019. The samples consisted of 98 patients with diabetes chosen through available sampling and randomly assigned into study and control groups. For data collection, diabetes quality of life (DQOL) short form clinical questionnaire, Persian version, was used. The intervention involved training self-management conducted through 60 sessions via a designed weblog. The obtained information was introduced into SPSS 21, and analyzed through Mann-Whitney, t-test, and paired t-test.

**Results:**

According to the results of this study, the mean age of the examined patients was 32.1 ± 4.9 years, where the major participants were male (*n* = 52 in the test group, 52.5%). The results showed that after the intervention, the test and control groups were different in terms of anthropometric variables and metabolic indicators; the mean waist circumference in the test and control groups was 98.6 ± 9.8 and 101.5 ± 7.8, respectively; the mean FBS following the intervention in the test and control groups was 131.08 ± 16.04 and 238.2 ± 40, respectively; and the mean BMI postintervention in the test and control groups was obtained as 27.3 ± 3.4 and 30.1 ± 3.8 respectively, where these differences were significant according to independent t-test (*p* < 0.05). The mean score of quality of life postintervention in the test and control groups was obtained as 56.1 and 49.9 respectively; according to Mann-Whitney test, the difference between the two groups was significant (*p* < 0.05).

**Conclusion:**

The results of the present study revealed the positive effect of weblog based self-management on the quality of life of patients with diabetes following the intervention. Further, reduced levels of FBS, BMI, as well as systolic and diastolic blood pressure were also observed, which could be due to increased awareness of patients about their abilities, its risks, as well as the ways to control and treat it.

## Background

### Diabetes

Diabetes is one of the most important chronic disease (long-lasting and incurable), which has a very high prevalence worldwide. It is categorized into three major groups: type 1 diabetes (T1D), type 2 diabetes (T2D), and gestational diabetes [1]. The international association has reported type 2 diabetes to account for over 90% of all diabetes cases. It is expected that the total number of patients with diabetes globally would grow in 2030 from 285 million to 439 million patients [2]. The ascending trend of the number of diabetic patients increases the need to improving both the treatment and care. The fact that the disease treatment and its associated factors are very complex again further increase the need to patient education and medical supervision [3–5]. Today, the high prevalence of diabetes is considered a major public health threat, where some of the adverse consequences and problems caused by this disease include diabetic foot ulcer and amputation along with cardiac and ophthalmology complications [6, 7].

### Self-management

So far, in addition to pharmacotherapy, various solutions have been presented to control the blood sugar levels. Many studies have indicated the positive effect of different interventions in controlling diabetes. The investigated interventions in these studies include lifestyle modifications, such as improving the nutritional status, increasing physical activity and quit smoking [8–11], controlling and following up patients through nurses [12], and performing self-care interventions as well as adopting interventions across the society include public health interventions [9–17]. One of the plans which has recently attracted the attention of researchers is the diabetes self-management. Self-management refers to an active process which is guided by the patient and includes special activities in order to achieve disease management goals [18]. The aim of diabetes self-management is to control blood sugar, prevent the acute and chronic complications, and enhance the quality of life of these patients. Generally self-management is an important method for keeping and improving the healthy behaviors and status of patients [19, 20].

The purpose of diabetes self-management is controlling blood sugar, preventing acute side-effects and increasing the quality of life of diabetic’s patients. Self-management is generally important method to preserve and improve the behaviors and health condition of patient [21]. Changes in these lifestyle factors may reduce the risk of T2D and influence the progression of this disease [22]. Many researches showed the association between dietary factors and the incidence of T2D, the associations between dietary behaviors/diet quality indices, food groups, single foods and beverages, alcohol, specific macro- and micronutrients and incidence of T2D. These findings could be of importance for the prevention of T2D [23]. Recent reports summarized evidence for selected dietary factors regarding prevention of T2D [24].

Exercise training improves glycemic control [25] exercise training also improves cardiovascular disease risk among people with type 2 diabetes [26, 27]. Current Australian guidelines recommend that people with type 2 diabetes or pre-diabetes accumulate a minimum of 210 min per week of moderate-intensity exercise or 125 min of vigorous-intensity exercise consisting of aerobic and resistance modes [28–31].

Self-regulation that enables a patient to exert confidence and control over their diet and exercise behaviors is a key component to effective lifestyle intervention adherence [32, 33]. One approach to enhance patient self-regulation is to provide them with immediate feedback based on the results of their behaviors [34]. Self-monitoring of health markers and behaviors beyond the clinical setting has been used as an effective tool to monitor treatment response and improve adherence for a variety of health outcomes including body weight, blood pressure and physical activity [34]. This suggests that self-monitoring of blood glucose (SMBG) in T2D could serve as an immediate feedback function to provide patients with evidence of the biological effect of lifestyle choices on blood glucose levels that may improve adherence to lifestyle prescription and improve glycemic outcomes [35–37].

### Diabetes self-management education

Diabetes self-management education (DSME) involves facilitating knowledge, skills, and their abilities required for self-care in diabetic patients. Diabetes self-management support (DMSM) refers to the support required for implementing and sustaining coping skills and behaviors that are constantly required for self-management in diabetic patients [38–41]. Self-care interventions of diabetic patients indicate advantages in terms of quality of life [42, 43] as well as glycemic control [44–46]. However, low participation [47–50], the effect of transition over time [51], and access to well-trained experts to support self-management are considered limitations [52, 53].

### Quality of life

Quality of life is considered an important outcome of health, and is of interest as a major issue in taking care of different patients including those with diabetes. The reason is that one of the major complications of diabetes is its adverse effect on quality of life of these patients. According to the World Health Organization (WHO) recommendations, quality of life refers to the personal perception of a person about their life situation concerning the culture and value system of the society as well as its relationship with the goals, expectations, standards, and needs [53 (para 1–5), 54, 55].

### Web based self-management (in diabetic patient)

One of the ways through which self-management can be encouraged in individuals and in turn the quality of life of patients with diabetes has increased is use of social networks. Application of web-based self-care interventions is promising, since they provide easy access for patients with computer literacy, and can be accomplished by spending minimum costs [56]. Web-based media have improved patient knowledge, the extent of behavioral change, and clinical outcomes for a wide range of conditions [57].

Investigations on patients with diabetes suggest the positive psychological and clinical effects including HbA1c and weight using websites [57]. In addition, further use of websites has been associated with greater clinical improvement: the users experienced greater reduction in HbA1c through websites [58].

In a review study conducted by Cooper and Kar (2014) in England titled “a new start: the role of social media in diabetes education”, it was shown that self-care is an integral part of diabetes management and those with diabetes, by daily life in this condition, improve their skill in self-management. Internet has become a valuable resource for diabetic people; for example, social media, weblogs and help sites for self-effective patients, let them to increase and improve the content of site, share their experiences with others and communicate with others with similar conditions [59].

In a study by Mohammadi et al. (2019) regarding “The effect of self-care education through social networking on quality of life in type 1 diabetic patients in Sanandaj, Iran” the results showed that before the intervention, the mean quality of life score in the intervention group was 40.82, but after the intervention, it was 34.33, which was not statistically significant (*p* = 0.638), but also in the control group There was no significant difference between the quality of life scores before and after the intervention (*p* = 0.6147) [60]. In a cohort study by Yu et.al (2014) in Toronto titled “web-based intervention to support type 2 diabetes patients’ self-management: effect on self-efficacy, self-care and discomfort”, the results showed that self-management website has not improved the self-efficacy of diabetic patients and the use of website was limited [61].

Diabetes based self-management includes five stages: regular measurement of glucose, regular purchasing and consumption of drugs, selecting suitable diet, exercise, and adhering to the physician’s orders and prescriptions [62].

### Scope

Based on the mentioned points and in spite of the numerous problems of the operative patients along with the development of chronic complications and various disabilities as well as staggering costs and the progressive increase in morbidity and mortality of diabetes both in developing and developed countries, and as sparse studies have dealt with investigating the effect of weblogs on the quality of life of diabetic patients, and concerning the availability of Internet websites and networks, the aim of the present study was to determine the effect of self-management education using weblogs on the quality of diabetic patients in Kermanshah City.

## Methods

### Participants

This research is an interventional study performed on patients referring to Taleghani diabetes clinic in Kermanshah in winter 2018 and spring 2019.

The statistical population of the research consisted of diabetic patients referring to the above-mentioned center, chosen through available sampling and randomly assigned into two groups. The number of test samples in this research according to the study by Saeidpour et al. (2013), significance level of 0.05, test power of 80%, mean difference between the two groups as 10, standard deviation in the intervention group as 17.5, standard deviation in the control group 15.8, the number of samples recorded for each group was determined as 44. Considering 10% attrition, the number of samples for each group was estimated at 49 and 98 in total [63].

$$ n=\frac{{\left({Z}_{1-\frac{\propto }{2}}+{Z}_{1-\beta}\right)}^2\kern0em \left({\sigma_1}^2+{\sigma_2}^2\right)}{{\left({\mu}_{1-}{\mu}_2\right)}^2} $$

These samples were chosen through available sampling, who were randomly assigned into study and control groups. In this way, each group accommodated 49 diabetic patients. Inclusion criteria were having diabetes for at least 5 years, having literacy to use weblog and intention to cooperate. The exclusion criteria included a lack of follow-up of the training program and an unwillingness to continue to participate in the study were not conforming to education program and non-intention to participate in the study.

### Ethics

During the research process, the Helsinki Research Ethics Statement was followed [64]; also this study has been approved by the research ethics committee of Kermanshah Medical Sciences University with code IR.KUMS.REC.1397.1041.

### Treatment (intervention) and education

After allocation of the patients into intervention and control groups through random assignment, their information was extracted from the diabetes Center of Taleghani hospital in Kermanshah city. Then, through the contact number as well as the social networks, the necessary notifications were performed about the weblog, the way of access and using its contents to be educated, and then education was performed for the intervention group through a weblog (http://diabeticmanagment.blogfa.com). In this weblog, the self-management education five stage approach was used which involved regular measurement of glucose, regular prescribe and consumption of drugs, selecting suitable diet, exercise, and adhering to the physician’s commands and prescriptions [62].

The self-management education was performed for 20 weeks (5 Month) for the intervention group; educational content was placed in the weblog 3 days a week, and in each session as long as 1:30 h [61].

**Fig. 1:**
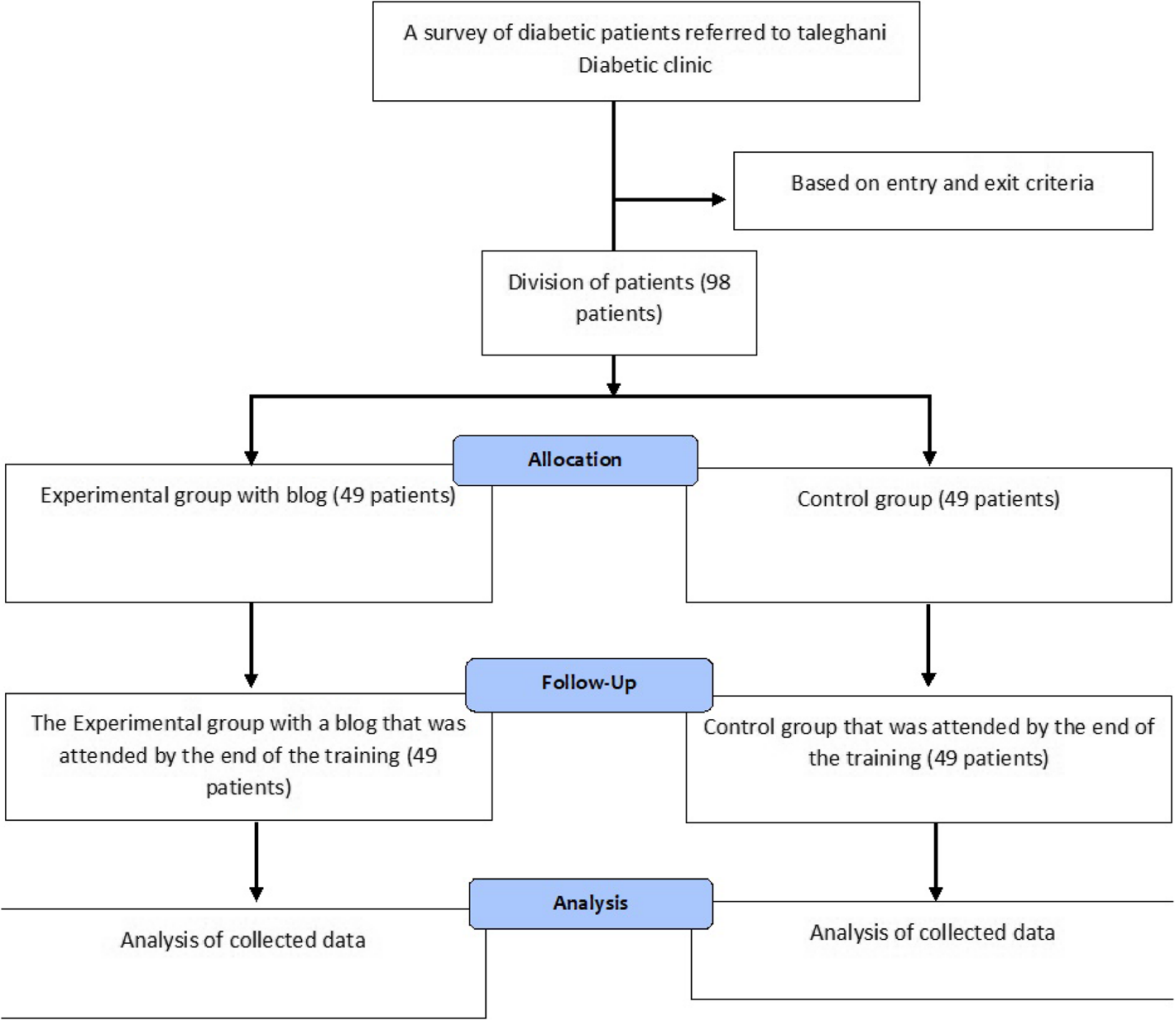
The study process (CONSORT 2018 Flow Diagram – Diabetic patient)

Overall, 60 sessions were predicted for educating diabetic patients in this way. Specifically, twenty sessions were posted in the weblog regarding selecting suitable diet as text, video, recorded voice, and the shape of the proper nutritional pyramid for diabetic patients, and they received the necessary education. Then, twenty sessions related to suitable exercise (exercise-related blog content) involved two parts of aerobic exercise and physical activity, such that the patient allocated four sessions per week, each session lasting 45 min to exercise. Further, ten sessions were dedicated to regular consumption of the prescribed drugs; drugs including insulin or antidiabetic drugs prescribed for the patients. Regular consumption of them was educated, and the complications of diabetes were also provided to the patients as educational clips and films. Furthermore, the proper way of keeping and injecting insulin was also covered. Then five session were dedicated to regular measurement of glucose, recording it, and presenting it to the physician. Furthermore, the way of using digital glucometer was also trained. In addition, five session were allocated to adhering to the physician orders and prescriptions, and finally one session was dedicated to concluding the contents and points (Fig. 1).

### The study process

#### Measures

The educational content that was posted in the weblog included educational clips, voices recorded in Persian, written text, images, and educational posters related to each of the five stages of the diabetes based self-management approach. These contents were posted in the weblog given the needs of the study groups as well as the extent of self-management that had occurred in them [65]. In case the patients had any question and generally in order to answer the questions, the contact number as well as email of the researchers was provided to the patients in the weblog.

On the other hand, for the control group no intervention was done by the research team. They only received routine interventions from the diabetes Center including educational programs, controlling nutrition, and constant blood sugar monitoring. By the end of the education, the test questionnaire was again completed by the two groups and the effect of education was measured.

#### Questionnaire

For collecting data about quality of life, diabetes quality of life short form clinical questionnaire (DQOL), Persian version, which has been extensively used in testing the quality of life of diabetic patients in Iran [66] was used. Further, to collect the background information and demographics, a researcher made demographic information form was utilized.

The demographic information of individuals was captured through the checklist, while the information associated with investigating the quality of life was completed through the Persian DQOL in the research for both groups through self-report.

DQOL questionnaire consists of 60 items, whose reliability and validity were first calculated by Thomas E. Burroughs in 2004, and was extracted as 15 reduced items. This questionnaire is used for investigating type I and type II diabetic patients, and captures the dimension of care behaviors of the patient and satisfaction with the disease control. In the study performed by Nasihatkon et al. (2012) regarding “determination of the reliability and validity of DQOL short form clinical questionnaire in Persian”, its validity was confirmed through content validity method. Further, its internal consistency was 0.77 via Cronbach alpha method [67]; the range of 15–75 for scores related to the response of the questions of the scoring method as absolutely dissatisfied (score 1), dissatisfied (score 2), average (score 3), satisfied (score 4), and absolutely satisfied (score 5), were used in the statistical analysis and the quality of life of patients was categorized in line with that score. Based on the total score, they were categorized into three groups: unfavorable (score less than 30), relatively favorable (31–60), and favorable (greater than 60).

#### Statistical test

After completing questionnaires by the studied sample and introduced data into SPSS-21 software, the results were reported in the descriptive statistics format (frequency, mean and standard deviation) and analytical statistics. Tables and diagrams were used to describe the qualitative variables and central tendency and dispersion indicators used for quantitative variables. The normality of data was measured by Kolmogorov-Smirnov test, and then, with Independent sample t-test, paired t-test, Chi-square, Fisher’s exact test used.

## Results

Relative frequency distribution and demographic variables in research units, it was reported that the most participants in the study were male (52 = 52.5%), with below diploma education (83 = 83.8%), married (86 = 86.9%), without special illness (60 = 60.6%), employed (50 = 50.5%), living in city (87 = 87.9%), owner (72 = 72.7%), smoking history (76 = 76.8%), lack of exercise (73 = 73.7%), no history of stress, depression and taking antipsychotic medication (91 studies = 91.9%; 72 studies = 72.7%, 81 studies = 81.8%), respectively. Sixty-one subjects believed that their relationship with their spouses is satisfactory and 63 studies had more than 5 million Rial monthly income (Table 1).

**Table 1:** Relative frequency distribution and demographic variables in the research units

		Percent	Frequency
Sex	Male	52.5	52
	Female	47.5	46
	Total	100.0	98
Education	less than Diploma degree	83.8	83
	more than Diploma degree	16.2	15
	Total	100.0	98
Marriage	Married	86.9	86
	Single and divorced	13.1	12
	Total	100.0	98
special disease	No	60.6	60
	Yes	39.4	38
	Total	100.0	98
Occupation	Employee	50.5	50
	Other job	49.5	48
	Total	100.0	98
Residency	Urban	87.9	87
	Rural	12.1	11
	Total	100.0	98
Housing situation	Own house	72.7	72
	Tenant	27.3	26
	Total	100.0	98
Cigarette smoking	No	23.2	22
	Yes	76.8	76
	Total	100.0	98
Exercise	Yes	26.3	25
	No	73.7	73
	Total	100.0	98
Stress	Yes	8.1	7
	No	91.9	91
	Total	100.0	98
Depression	Yes	27.3	26
	No	72.7	72
	Total	100.0	98
Antipsychotic medication	Yes	18.2	17
	No	81.8	81
	Total	100.0	98
Exposure to smoke	Yes	8.1	7
	No	91.9	91
	Total	100.0	98
Relation between spouse	Very satisfied	15.2	14
	Satisfied	61.6	61
	Normal	13.1	13
	Dissatisfied	7.1	7
	Very dissatisfied	3.0	3
	Total	100.0	98
Monthly income money by Iranian Rial	Less than 5 million (Rial)	36.4	35
	More than 5 million (Rial)	63.6	63
	Total	100.0	98

The relative and absolute frequency of the gender of the participants in this study in terms of the test and control groups regarding gender (*p* = 0.27), place of residence (*p* = 0.20), accommodation status (*p* = 0.23), smoking (*p* = 0.10), exercise (*p* = 0.07), stress (*p* = 0.06), depression (*p* = 0.77), drug consumption (*p* = 0.96), exposure to cigarette smoke (*p* = 0.44), relationship between couples (*p* = 0.10), academic status (*p* = 0.55), marital status (*p* = 0.12), having special diseases (*p* = 0.90), occupation (*p* = 0.91), and income status (*p* = 0.93) showed no significant difference..

Elevated blood pressure is when readings consistently range from 120 to 129 systolic and less than 80 mmHg diastolic, Hypertension Stage 1: is when blood pressure consistently ranges from 130 to 139 systolic or 80–89 mmHg diastolic, Hypertension Stage 2: is when blood pressure consistently ranges at 140/90 mmHg or higher, Hypertensive crisis: If your blood pressure is higher than 180/120 mmHg. According to the results in Table 2 revealing the mean rank of the test and control groups in terms of the variables of interest in the study, based on the Mann-Whitney test findings, a significant difference was observed between the test and control groups before and after the intervention in terms of the variables of mean systolic and diastolic blood pressure after the intervention (*p* < 0.05).

**Table 2:** The difference of the mean and standard deviation as well as the mean score of test and control groups before and after the intervention in terms of the variables of interest in the study

	group	N	Mean Rank	Mean	Std. Deviation	*p*-value
age	case	49	44.63	31.36	5.29	0.06
	control	49	55.26	32.98	4.42	
Systole (Before)	case	49	49.38	134.89	18.58	0.82
	control	49	50.61	135.20	19.05	
Diastole (Before)	case	49	52.57	73.06	13.26	0.35
	control	49	47.48	72.30	10.36	
Systole (After)	case	49	48.03	118.70	15.14	0.001
	control	49	51.01	120.71	13.54	
Diastole (After)	case	49	49.56	60.91	6.28	0.04
	control	49	51.45	61.72	10.06	

Test statistic: Mann-Whitney test (*P* < 0.05)

According to the results in Table 3 capturing the mean difference of the studied variables in terms of the test and control groups, it was found that following the intervention, the mean waist circumference in the test group was 98.6 ± 9.8, 101.5 ± 7.8 in the control group, the mean FBS was 131.08 ± 16.04 and 238.02 ± 40.01 in the test and control groups respectively after the intervention, and the mean BMI was 27.3 ± 3.4 and 30.3 ± 3.8 in the test and control groups respectively after the intervention. Based on the findings obtained from independent t-test, there was significant difference between the two groups in terms of waist circumference, BMI, and FBS after the intervention (*p* < 0.05).

**Table 3:** Investigating the mean difference of the studied variables in terms of test and control groups before and after the intervention concerning the variables of interest

	group	N	Mean	Std. Deviation	*p*-value
Waist (Before)	case	49	100.6	9.83	0.06
	control	49	99.90	10.17	
Waist (After)	case	49	98.68	9.82	< 0.0001
	control	49	101.58	7.82	
FBS (Before)	case	49	250.26	50.55	0.18
	control	49	252.06	39.58	
FBS (After)	case	49	131.08	16.04	< 0.0001
	control	49	238.24	40.01	
BMI (Before)	case	49	29.77	3.66	0.42
	control	49	29.64	4.03	
BMI (After)	case	49	27.30	3.45	< 0.0001
	control	49	30.16	3.89	

Test statistic: independent t-test (*P* < 0.05)

According to the results in Table 4 capturing the mean difference of the studied variables in terms of the test and control groups, it was found that following the intervention, the mean waist circumference in the test group was 98.6 ± 9.8, 101.5 ± 7.8 in the control group, the mean FBS was 131.08 ± 16.04 and 238.02 ± 40.01 in the test and control groups respectively after the intervention, and the mean BMI was 27.3 ± 3.4 and 30.1 ± 3.8 in the test and control groups respectively after the intervention. Based on the findings obtained from independent t-test, there was significant difference between the two groups in terms of waist circumference, BMI, and FBS after the intervention (*p* < 0.05).

**Table 4:** Investigating the difference of mean and standard deviation of the studied variables across the test groups before and after the intervention as well as in the control group before and after the intervention

group			Mean	Std. Deviation	*p*-value
case	Pair 1	FBS (Before)	250.26	50.55	< 0.0001
		FBS (After)	131.08	16.04	
	Pair 2	Waist (Before)	100.6	9.83	< 0.0001
		Waist (After)	98.68	9.82	
	Pair 3	BMI (Before)	29.77	3.66	< 0.0001
		BMI (After)	27.30	3.45	
	Pair 4	Systole (Before)	134.89	18.58	< 0.0001
		Systole (After)	118.70	15.14	
	Pair 5	Diastole (Before)	73.06	13.26	< 0.0001
		Diastole (After)	60.91	6.28	
control	Pair 1	FBS (Before)	252.06	39.58	0.89
		FBS (After)	238.24	40.01	
	Pair 2	Waist (Before)	99.90	10.17	0.65
		Waist (After)	101.58	7.82	
	Pair 3	BMI (Before)	29.64	4.03	0.98
		BMI (After)	30.16	3.89	
	Pair 4	Systole (Before)	135.2	19.05	0.431
		Systole (After)	134.9	15.1	
	Pair 5	Diastole (Before)	72.30	10.36	0.22
		Diastole (After)	61.72	10.06	

Test statistic: paired t-test (*P* < 0.05)

Based on the findings of Table 5 and paired t-test, the mean and standard deviation of FBS was 254.2 ± 50.5 and 251.08 ± 16.04 before and after the intervention in the test group respectively, the mean waist circumference was 102.6 ± 7.8 and 100.6 ± 7.8 before and after the intervention respectively in the test group, the mean BMI was 30.3 ± 3.6 and 28.3 ± 3.4 before and after the intervention respectively in the test group, the mean systolic blood pressure was 134.18 ± 8.5 and 120.7 ± 13.5 before and after the intervention in the test group respectively, and the mean diastolic blood pressure was 73.13 ± 06.3 and 62.9 ± 6.2 before and after the intervention respectively in the test group. According to the findings of paired t-test, the difference between and after the intervention was significant for the test group (*p* < 0.05).

**Table 5:** The mean score of quality of life of patients with diabetes before and after the intervention in terms of test and control groups

	group	N	Mean Rank	Sum of Ranks	Mean	Std. Deviation	*p*-value
Quality of Life before	case	49	49.06	1914.00	37.8	3.6	0.08
	control	49	50.72	3036.00	38.5	3.9	
Quality of Life after	case	49	56.14	2751.00	59.1	2.2	0.03
	control	49	50.21	2199.00	56.6	2.8	

Test statistic: Mann-Whitney and Wilcoxon test (*P* < 0.05)

According to Table 5 and investigation of the mean score of quality of life of patients with diabetes before and after the intervention, before the intervention the mean score in the test and control groups was 37.8 and 38.5 respectively, where according to Mann-Whitney test, the score differences were not significantly different between the two groups (*p* = 0.089). The mean quality of life score after the intervention in the test and control groups was obtained as 59.1 and 56.6 respectively, where according to Mann-Whitney test, the score difference was significant between the two groups (*p* < 0.05). Furthermore, the difference of the scores before and after the intervention in the test and control groups according to Wilcoxon test showed a significant difference, suggesting improved quality of life after the intervention in the test group. Finally, according to quality of life questionnaire scoring, a relatively desirable quality of life was found in patients post intervention.

## Discussion

Using the resources and social networks has positive effect on increasing the health and awareness of people, especially patients, in the society and this process provides enough information to take an effective step in controlling diabetes better. Therefore, the accesses of diabetic patients to the required information help them to take the proper decision and have higher control on the environment [68]. Education of preserving and promoting health is one of the initial approaches to help people to change their wrong habits that are implemented in the widespread and diverse levels of the society [69]. Today, information and communication technology is used as a powerful tool to promote the quality and efficiency of education such that it has transformed the traditional education methods [70]. The rapid development of internet technologies has caused that electronic education becomes an important form of education in the information era [71]. Electronic education is a widespread set of applied software and information technology based method including computer, compact disk, internet network, intranet and virtual university that provide the life-lasting education for people in each time and place [72]. The results of this study shows that based on the results of this study, most participants in the study were male, with below diploma education, married, employed and the mean rank of quality of life after intervention was significant in the experimental and control groups.

Diabetes is the main cause of retinopathy, neuropathy, nephropathy, and the cause of 60% of feet amputation cases [73]. Further, diabetes increases the risk of heart attacks, strokes, and the mortality caused by cardiovascular disease by 2–4 times compared to other patients [74]. Therefore, investigating the quality of life and enhancing it in diabetic patients have always been considered an important health outcome, and is noted as a major issue in taking care of patients with diabetes [59]. In this regard, various studies have dealt with investigating the quality of life of diabetic patients. In a review study conducted by Sheps et al., it was found that weblogs, microblogs, social network sites, professional network sites, and thematic network sites have had maximum applications in healthcare [70]. In another review study performed by Cooper and Kar, it was reported that self-care is an indispensable part of diabetes management, and diabetic individuals through daily living with this condition improve their skill regarding self-management. This study suggests that Internet has changed into a valuable resource for diabetic patients. Indeed, Internet allows them to promote and improve the site content, share their experiences with others, and communicate with other individuals in a similar situation [75]. Further, in the study by Mano, a promising theoretical framework was presented for effectiveness of use of social media in encouraging use of online healthcare services. They reported that finding practical and economical solutions to support the use of social media and encouraging access to online health information and usage of online healthcare services can enhance the health literacy and health self-management at individual level as well as productivity in presenting healthcare services at institutional level [76, 77].

Soleimani et al. [78] reported that concerning the effect of diabetes control and self-care behaviors as well as self-management on the desirability of quality of life, it is proposed that these educations be taken more seriously for enhancing the quality of life of diabetic patients, which highlight its importance. Similarly, Ghiathvandian et al. [79] also reported that self-management education was effective on the quality of life of diabetic patients and recommended that concerning the important role of education in the management and control of chronic diseases such as diabetes, more efficient and effective self-management educations should be noted by health policymakers regarding diabetic patients. In the study by Saeidpour et al. [80] again self-care and self-management educations were recommended for enhancing the quality of life of diabetic patients, and considered it as a public health promoter.

Such educations have found a new form considering the progressive growth of use of social networks and weblogs, which can be more effective and available for all patients [81]. The epidemics of use of Internet and social networks is such that all people even in the farthest points of the country can also gain access to the scientific experience and concepts of physicians and researchers in healthcare issues, and gain more awareness about their disease [81]. In this way, anyone especially patients who have been focused on in this research in particular can create a personal weblog through the Internet easily and within a short time. Alternatively, they can become members of different weblogs and websites and see or publish the contents they have produced including text, image, voice, and video [81]. Accordingly, this highlights the importance of self-management education through weblogs on the quality of life of diabetic patients.

In this study, the self-management education through weblog resulted in improvement of anthropometric indicators including the waist circumference as well as BMI and FBS. In investigating the mean difference of the tested variables in terms of the test and control groups, it was observed that after the intervention, the waist circumference, FBS, and BMI had a significant difference between the test and control groups. Further, paired t-test results revealed significant difference before and after the intervention in the test group in terms of the criteria including FBS, mean waist circumference, mean BMI, as well as mean systolic and diastolic blood pressure.

The results of this study also suggested the positive effect of self-management education through weblog on improving the quality of life of diabetic patients. According to the results, it was also reported that the mean quality of life score of patients before the intervention was 49.06 and 50.7 in the test and control groups, respectively, showing no significant difference. However, in investigating the quality of life post intervention in the two groups, it was observed that the quality of life after the intervention was increased in the test groups, respectively revealing a significant difference. This indicates that the quality of life of patients has improved after the intervention. The positive effect of the intervention for the patients in the study by Gheiathvand et al. [79] along with Saeidpour et al. [62] was also reported suggesting that the difference between the quality of life score of patients before and after the intervention was significantly different. Baghianimoghadam et al. [82] again in their study reported that educational intervention caused enhanced quality of life of diabetic patients after the intervention. Such a positive effect was also reported in the study by Aghamolaei et al. [83] as well as Balenjani et al. [84]. The study by Murray et al. [85] also reported the positive effect of web-based education on enhancing the level of awareness of patients and improved self-management in these patients. They suggested that these educations can improve and enhance the level of quality of life of diabetic patients.

In the study by Nundy et al., it was found that based on investigating the diabetes cell phone project to improve glycemic control and save costs for the participants in a program in Chicago, improvement in glycemic control and patient satisfaction with general care had a significant difference; notably, 8.8% was saved in the net healthcare costs. This study also reported that the healthcare plans can support the three goals of improving patient health, improving the public health, and reducing the healthcare per capita costs [75].

In the study by Kate Lorig et al., it was reported that online self-care program of diabetes for patients were followed up for 18 months indicated that after 6 months of study, physical activity and self-efficacy improved significantly for the test groups compared to the control group who received routine care. However, no change was found in other behavioral or health indicators [66].

In a cohort study performed by Yu et al. (2014) in Toronto on “web-based intervention for supporting self-care among patients with type II diabetes: effect on self-efficacy, self-care, and diabetes”, it was found that after 9 months, the self-efficacy score and the clinical outcomes did not improve, where the self-management website did not improve the self-efficacy situation in diabetic patients and usage of website was limited and thus had no significant effect on the self-management of diabetic patients [64]. This research reported there was a significant difference between experimental and control groups in terms of stress. Further, in the study by Hoffman et al. [86] it was reported that the Internet based self-care interventions may lead to diminished anxiety associated with diabetes among individuals with type II diabetes, and generates positive outcomes for the psychological and behavioral situation of adults with type II diabetes. They can also improve dietary habits, behavioral habits, and medical management in these patients. The results of the study by Murray et al. [85] on investigating web-based self-care also suggested positive effects for the patients.

These results indicate the importance of self-management training in diabetic patients and improve the status of stress and its complications in patients. The results of our study showed that the BMI of the Experimental group after intervention was significantly decreased, which was confirmed in a study by Tahir et al. (2017), and diabetic patients participating in self-care education program BMI and their weight decreased significantly [87]. According to the study, Horner et al. (2019) showed that self-care education had a significant effect on quality of life and BMI of asthmatic patients [88]. The results of Singh et al. (2019) have shown that BMI in diabetic patients with self-care education has significantly decreased [89]. Which is consistent with the results of the present study. The present study has shown that self-care education has been effective in reducing blood pressure in diabetic patients, which confirms our study, Simanjuntak (2019), that self-efficacy education has been effective in reducing systolic and diastolic blood pressure in diabetic patients, [90]. Also, Zimbudzi (2018) review study has shown that self-care education has a significant effect on systolic blood pressure in diabetic patients and has decreased significantly in the control group [91]. This is in agreement with the results of this study. A study by Feng et al. (2018) has shown that systolic blood pressure significantly decreased in diabetic Chinese patients, in intervention group after self-care education [92]. In a study conducted by Hailu et al. (2018) in Ethiopia, diabetic patients in the intervention group received a significant reduction in FBS, systolic blood pressure and diastolic blood pressure in the control group after intervention [93]. This is in agreement with the results of the present study. In confirmation of the results of this study, Kisokanth et al. (2019) showed that self-care education in diabetic patients improved the FBS and BMI status of the patients in the intervention group compared to the pre-intervention group [94]. According to the results of this study, the study of Jiang et al. (2019), WILMOTH (2019), showed that self-care education in diabetic patients improved weight, waist circumference and quality of life in diabetic patients [95, 96].

In this study, only 20–47 years old people with the ability of using internet and having access to internet were considered. Regarding the electronic education and the absence of research team supervision, the partial implementation of self-management was another limitation of this study. This study was also done on the diabetic patients visiting Taleghani diabetes clinic and patients visiting other healthcare wards did not include in the study. Future studies are expected to have no limitations to the present study and be tailored to the more valid questionnaires in different languages.

## Conclusion

The results of the present study suggest the positive effect of weblog based self-management on enhancing quality of life, reducing the levels of FBS, BMI, and systolic as well as diastolic blood pressure across the intervention groups, which can be due to increased awareness of diabetic patients about its risks as well as the ways to control and treat it. Raising patients’ awareness through social networks, giving them better access to new medicines, care and methods.

Concerning the increasing application of the Internet and social networks, weblogs can change into one of the best digital media for educating diabetic patients to be further noted by health policymakers. The reason is that it is a novel and efficient educational method and can both reduce the healthcare costs and make them more economical.

## Data Availability

Datasets are available through the corresponding author upon reasonable request.

## Abbreviations

BMI: Body Mass Index
DMSM: Diabetes Self-Management Support
DQOL: Diabetes Quality of Life
FBS: Fasting Blood Sugar
HbA1c: Hemoglobin A1c
WHO: World Health Organization
